# Polylactic Acid Bioplastics (PLA BPs): A Threat to the Reproductive Health of the Mediterranean Mussel *Mytilus galloprovincialis*

**DOI:** 10.3390/jox16050176

**Published:** 2026-09-17

**Authors:** Mariachiara Galati, Giuseppe De Marco, Mariano Dara, Giulia La Pusata, Maria Giovanna Parisi, Matteo Cammarata, Maria Maisano, Tiziana Cappello

**Affiliations:** 1 Department of Chemical, Biological, Pharmaceutical and Environmental Sciences, University of Messina, 98166 Messina, Italy; mariachiara.galati@unime.it (M.G.); giuseppe.demarco@unime.it (G.D.M.); giulia.lapusata@studenti.unime.it (G.L.P.); maria.maisano@unime.it (M.M.); 2 Marine Immunobiology Laboratory, Department of Earth and Sea Sciences, University of Palermo, 90123 Palermo, Italy; mariano.dara@unipa.it (M.D.); mariagiovanna.parisi@unipa.it (M.G.P.); matteo.cammarata@unipa.it (M.C.); 3 Universal Scientific Education and Research Network (USERN), Italy

**Keywords:** bioplastics, bivalves, reproductive toxicity, gonads, sex-related effects, tissue morphology, d-PAS/PAS reactions, NMR metabolomics, bioenergetic metabolism, detoxification pathways

## Abstract

In the context of the global transition towards a more sustainable economy, the use of bioplastics (BPs) is rapidly increasing across several sectors, including industry, medicine and cosmetics. Among BPs, polylactic acid (PLA) is the “polymer of the 21st century”. Because of its wide use and slow degradation capacity, it can be found in various aquatic matrices, causing potential health concerns for biota. This work evaluated the influence of environmental concentrations (0.5 µg/mL) of PLA BPs on the reproductive health of the Mediterranean mussel *Mytilus galloprovincialis*, following a short-term exposure of 7 days. This resulted in an early spawning event from the gonads of both sexes, accompanied by an intense hemocyte recall, suggesting the onset of a pro-inflammatory condition. Alterations in the bioenergetic pathway affecting glycogen reserves as an adaptive strategy were revealed in both sexes by proton Nuclear Magnetic Resonance (^1^H NMR)-based metabolomics, in addition to disorders in the redox balance. This latter was more evident in males, as confirmed by the increased activity of antioxidant (catalase) and detoxifying (glutathione *S*-transferase) enzymes, while no lipid peroxidation occurred. Overall, this study highlights the threat of PLA BPs on the reproductive system of non-target organisms, raising further questions on the reproductive fitness and survival of a species with high ecological and economic value.

## 1. Introduction

Today, plastics continue to play an essential role in our daily life [1]. However, scientific research has demonstrated the ability of various types of plastics to interact with and accumulate in biota, triggering dangerous consequences at the physiological, reproductive and behavioral levels [2,3,4,5,6,7,8]. This has led to a transition from conventional to eco-friendly polymers [9], also known as bioplastics (BPs), since they are considered equal and usable in the healthcare, pharmaceutical, electronic and packaging sectors [10,11,12]. This is a choice that would entail a great economic incentive since, currently, the disposal of conventional plastics involves considerable energy and financial expenditure, with a high release of waste products into the environment, such as greenhouse gases and bioavailable particulate [13,14,15]. However, the manufacturing of BPs has a low environmental impact since their production derives from vegetable raw materials [16,17].

Among the most promising BPs, polymers based on polylactic acid (PLA) are the most used. Indeed, PLA has been labeled as the “polymer of the 21st century”, occupying approximately 30% of production in 2021 and foreseeing a growth rate of over 40% for the next four years, with a turnover of millions of dollars [10,13,14,18,19,20,21]. PLA is an aliphatic polyester formed by lactic acid monomers conjugated linearly by ester bonds [22,23]. Its production depends on the microbial fermentative activity (i.e., *Lactobacillus*, *Lactococcus*) of highly sugary raw materials (i.e., sugar cane, corn, milk) [24]. The biodegradability of these polymers by specific microorganisms under particular chemico-physical conditions [25,26], resulting in the production of biomass, carbon dioxide and water [27], represents an excellent advantage for the ecosystem. Although this scenario is potentially present in nature [28,29], in marine environments certain factors can drastically affect this process. In fact, a limited presence of specialized microbial communities, coupled with low temperatures and reduced oxygen availability, may significantly slow down the hydrolytic process of BPs, leading to a delayed release of PLA oligomeric/monomeric units [30,31]. This, combined with the poor mitigation capacity of wastewater treatment plants (WWTPs), as already reported for conventional plastics [32], has led to the detection of BPs at concentrations of μg–mg/g in sewage sludge and marine sediments, and μg–mg/L in a variety of aqueous matrices [33]. In detail, in typical wastewater, reservoirs, and marine environments, current measured concentrations of biodegradable microplastics (including PLA) generally span between 0.054 and 180 μg/L [34], whereas environmental modeling based on the rapid multi-fold market increase in BPs estimates that environmental doses of these degradation products could surge to between 0.57 and 43.82 mg/L if waste management systems do not adapt [35]. Therefore, given their persistence in natural environments and future projections, PLA BPs may constitute a new class of emerging micropollutants [34,35,36,37,38].

Interestingly, organisms appear to ingest PLA preferentially over conventional petroleum-based polymers [39]. As a consequence, abnormalities in growth, histopathological damage and microbiota imbalances were documented in the digestive system of various fish species challenged by PLA BPs [39,40]. In zebrafish, studies comparing the effect at the sub-cellular level of BPs with those of conventional plastics (synthetic polymers derived from petrochemical products) have reported alterations in mitochondrial function, increased production of reactive oxygen species (ROS), and cell death [41,42]. Despite their similarities to conventional microplastics, the potential effects of PLA BPs on the reproductive system of biota remain poorly investigated. In zebrafish *Danio rerio*, exposure to PLA BPs caused disorders in the redox balance, differentiation of female germ cells and the production of hormones, with all these effects observed to be transmitted to offspring [43]. In invertebrates, the impact of PLA BPs on the reproductive system has been investigated in the roundworm *Caenorhabditis elegans* [44], in the ascidian *Microcosmus exasperatus* [45], and in the water flea *Daphnia magna* [36], with alterations in the cell cycle, increased mortality, and enhanced germline apoptosis.

This study was therefore designed to explore the effects of PLA BPs on the reproductive health of the marine bivalve *Mytilus galloprovincialis*. This is a native species of the Mediterranean coastal area subjected to intense anthropogenic pressure, including the discharge of plastic waste, which tends to accumulate in this semi-enclosed basin [45]. Due to its sessile lifestyle and filter-feeding behavior, *M. galloprovincialis* is widely used as model organism in environmental biomonitoring programs [3,46,47,48,49]. Its ability to filter large volumes of water and uptake and bioaccumulate particulate matter dispersed in the surrounding medium, including microplastics (MPs) and BPs, makes this species particularly suitable for investigating the biological effects of emerging micropollutants. Aware that this category of emerging micropollutants compromises the reproductive health of other aquatic species [43,44], the aim of this study was to evaluate the potential effects of PLA BPs of realistic and variable sizes (range 1 μm–>150 μm) at the environmental concentration of 0.5 µg/mL on gonads of both sexes of the non-target species *M. galloprovincialis* during a short-term exposure of 7 days. The selected concentration was determined based on data reported in the literature, obtained from field investigations [33]. After seven days of exposure, biomarkers of energy metabolism were measured in mussel gonads of both sexes by histomorphological, histochemical, and metabolomic approaches. Additionally, a pro-oxidant state and the related responsiveness attributable to the antioxidant and detoxifying system were investigated by biochemical assays evaluating the enzyme activity of catalase (CAT) and glutathione *S*-transferase (GST), respectively, as well as the occurrence of lipid peroxidation, in order to better understand the influence of sex on the modulation of biological responses to the same pollutant. The scientific hypothesis of this study was that sensitivity to PLA BPs in mussels might be higher in females, especially during the reproductive period, because of the high energetic cost of reproduction.

## 2. Materials and Methods

### 2.1. Polylactic Acid Bioplastics (PLA BPs)

The PLA material (NatureWorks^®^ Ingeo™ 2002D Extrusion Grade PLA) was kindly provided by the University of Palermo, Italy, in the form of a pellet. This was first mixed by mechanical action, and then passed through a stainless-steel sieve with graduated mesh to remove polymers larger than 150 μm. To establish the size of PLA particles, images were acquired by optical microscopy and analyzed using the open-source Fiji ImageJ software, release 2.18.0 [50,51]. From this evaluation, different sizes of PLA biopolymers (PLA BPs) were revealed, showing the highest percentage for the size range of 1–50 μm (78.85%), then 51–150 μm (17.90%), while the smallest amount was represented by PLA BP sizes bigger than 150 μm (3.25%).

The dispersion state and potential aggregation of the PLA particles under seawater conditions were not experimentally characterized. Therefore, the present study does not provide a direct assessment of changes in particle dispersion or aggregation during the exposure period, which cannot be excluded.

### 2.2. Acclimatization of Mussels and Experimental Design

Organisms of the Mediterranean species *Mytilus galloprovincialis* were collected in autumn (November) from the S.A.Co.M. (Società Allevatori e Commercio Molluschi, which means “Shellfish farming and Trade companies”) aquaculture plant located in the metropolitan city of Messina (Italy), and transported in fresh aerated seawater to the “Mesocosm Facility” of the Institute for Biological Resources and Marine Biotechnologies of the National Research Council (IRBIM-CNR; Messina, Italy). Following size selection (4–5 ± 0.4 cm valve length), a total of 20 organisms were randomly allocated to glass aquaria filled with 15 L of filtered seawater (FSW; filtered through a 4 μm filter, sterilized by UV 15 m^3^/h–40 mJ/cm^2^), provided by the IRBIM-CNR, a research center authorized by the Italian Ministry of Health as a “Facility for the use of animals for experimental purposes or for other scientific purposes” according to the Italian Legislative Decree 26/2014.

The acclimatization period lasted for a period of 15 days under controlled laboratory conditions of continuous aeration, salinity 35%, temperature at 18 ± 1 °C, pH 8 ± 0.02, and a photoperiod of L/D 12 h:12 h. The experimental plan was conducted in triplicate for evaluation of the cytotoxic effects of a single concentration of PLA BPs (NatureWorks^®^ Ingeo™ 2002D Extrusion Grade PLA) provided to mussels as a mixture of variable size (see Section 2.1). Specifically, only two experimental conditions were considered: a treated group (PLA BPs) with mussels exposed to 0.5 µg/mL PLA BPs, which was selected as an environmentally relevant concentration or future projection based on the literature data [33,34,35,36,37,38], and a control group (Ctrl), with mussels exposed only to FSW with no PLA BPs. Mussel exposure took place over 7 days, maintaining the same controlled laboratory conditions used for the acclimatization period. As they were in the form of an electrostatic powder, PLA BPs particles tended to adhere to the surrounding surfaces, and it was difficult to distribute them evenly in the exposure medium. Therefore, PLA BPs were first weighed in determined aliquots, and then each aliquot was suspended in known amounts of FSW and introduced in each aquarium in order to obtain the selected concentration of 0.5 µg/mL to be tested. This procedure was adopted to ensure that the correct amount of pollutant was accurately added to each aquarium. Water changes were performed every day until the end of the exposure, restoring the same experimental conditions in each group, and providing a commercial algae mix (Liquizell, Hobby, Dohse Aquaristik GmbH & Co. KG, Grafschaft-Gelsdorf, Germany) as food in drops, dosed according to the product specifications. After one week of exposure, 12 specimens were randomly sampled from each tank to have a statistically significant number of males and females, since the species does not show sexual dimorphism. From a total of nine individuals per sex (male; female) per experimental condition (Ctrl; PLA BPs), gonads were appropriately collected to carry out each planned analysis, since the amount of gonadal tissue from each individual mussel was enough for all the investigations. For histomorphological and histochemical analyses, tissue fragments of gonads were sampled from each organism, and promptly preserved in 4% paraformaldehyde (PFA; Immunofix, Bio-Optica; Milan, Italy), while for biochemical and metabolomic analysis, the rest of the gonadal tissue from each mussel was weighed, transferred in test tubes in liquid nitrogen for transport to the laboratory, and then stored at −80 °C.

### 2.3. Histological and Histochemical Analysis

The samples of gonads intended for histological and histochemical analysis (*n* = 9 per sex per experimental condition) were stored for 4 h in 4% PFA (37% formaldehyde diluted in phosphate-buffered saline, PBS, 1 M and pH 7.4; Immunofix; Bio-Optica, Milan, Italy) at 4 °C, followed by one rapid 10 min passages in 1 M PBS, and then dehydrated in an increasing series of ethanol (Mixetan; Alcoolital, Fossano (CN), Italy) (50°, 70°, 80°, 95°, and 100°). The samples were cleared with the organic solvent xylene (Sigma-Aldrich, St. Louis, MI, USA), first mixed with ethanol and then pure. Each tissue fragment was first treated with a mixture of xylene and Paraplast paraffin (Bio-Optica, Milan, Italy), and then followed two passages in paraffin alone, using an inclusion control unit (TEC2900; Histo-Line Laboratories, Pantigliate (MI), Italy). Manually, 4 μm thin sections were obtained using an automatic rotary microtome (MRS3500; Histo-Line Laboratories, Pantigliate (MI), Italy) and mounted onto microscope slides (two per slide). After drying overnight in an oven, each histological section was first deparaffinized in xylene and then rehydrated in a decreasing series of ethanol (100°, 95°, 80°, 70°, 50°, and 35°) to distilled water.

Some of the histological sections were stained with hematoxylin/eosin (H/E), as shown in [3], to determine the gonadal organization from which the sex and any potential alterations affecting the connective tissue and/or germinal cells could be revealed. The remaining sections were treated with diastasis–Periodic Acid Shiff/PAS (d-PAS/PAS) reaction according to the modified method reported by [52] in order to identify glycogen, a neutral polysaccharide positive for the oxidizing reaction of periodic acid and a Shiff reagent, to evaluate potential alterations affecting energy reserves in mussel gonads. In detail, one of the two sections on each slide was pre-treated with diastase to digest glycogen before d-PAS staining to serve as a negative control. The PAS-positive magenta staining was therefore attributed to the presence of glycogen. Both sections were then counterstained with hematoxylin and mounted for microscopic observation.

All images were obtained with a Zeiss Axio Imager Z1 microscope (Carl Zeiss AG, Oberkochen, Germany) equipped with immersion objectives and AxioCam digital camera (Carl Zeiss AG, Oberkochen, Germany) [53], with 40× magnification and exported in tiff format. Using the Image J (Image Processing and Analysis in Java) image software version 1.54i, the concentration of racemic polysaccharide detected by PAS reaction was calculated and reported as pixels [54,55,56]. Each histological and histochemical image has a scale bar of 20 μm.

### 2.4. Biochemical Analysis

To evaluate the antioxidant and detoxifying mechanisms of the enzymatic activity of catalase (CAT) and glutathione *S*-transferase (GST), respectively, and to verify the occurrence of any lipid peroxidative events through the content of malondialdehyde (MDA), a biochemical analysis was performed by the homogenization of 60 mg of individual male and female gonadal tissue, taken from mussels from the control and treated groups (*n* = 9 per sex per experimental condition). Gonadal samples were homogenized in a 0.1 M Tris-HCl buffer adjusted to pH 7.5, at a volume equivalent to 10 times the weight of the tissue in grams (total volume: 600 μL), using the homogenizer TissueLyser LT (Qiagen, Hilden, Germany) for 6 min with a cold rotor and stainless steel spheres at 50 oscillations/sec [3]. After centrifugation at 4 °C for 20 min (Centrifuge 5417R; Eppendorf, Milan, Italy) at 9000× *g*, the supernatant was aspirated and aliquoted into tubes for subsequent analyses, using the UV-mini 1240 spectrophotometer (Shimadzu, Milan, Italy). Each enzymatic analysis was performed at 25 °C [57], while the Pierce BCA Protein Assay Kit (Thermo Scientific, Waltham, MA, USA) was used to measure the total protein content, taking bovine serum albumin (Sigma-Aldrich, Munich, Germany) as a reference [58].

#### 2.4.1. Antioxidant and Detoxifying Activity

To measure any potential alterations in the redox balance at the cellular level, the activity of the enzyme CAT (μmol/min/mg of protein) was monitored, verifying the dismutation of hydrogen peroxide (H_2_O_2_) at a wavelength of 240 nm for 90 s [59].

In regard to the evaluation of the detoxifying system, the activity of the enzyme GST (nmol/min/mg of protein) was evaluated based on the generation of thioether, obtained from the conjugation of glutathione with 1-chloro-2,4-dinitrobenzene (CDNB). The reading was acquired after 3 min at a wavelength of 340 nm, as described by [60] (a total of nine samples for each condition).

#### 2.4.2. Lipid Peroxidation

To evaluate the degree of lipid peroxidation (LPO), the concentration of MDA (nmol/mg protein) was measured using the thio-barbituric acid reactive method (TBARS). In sealed glass tubes, 500 μL of thio-barbituric acid (Sigma-Aldrich, Munich, Germany) (0.0375% *w*/*v*), 500 μL of trichloroacetic acid (ITW Reagents, Monza, Italy) (15% *w*/*v*), 460 μL of distilled water and 40 μL of mussel gonadal sample were added sequentially. The mixture was kept in a hot bath at 90 °C for 15 min and read at a wavelength of 532 nm. 1,1,3,3-tetraethoxypropane was used to obtain the MDA calibration curve according to [61] (a total of nine samples for each condition).

### 2.5. Metabolomic Analysis

#### 2.5.1. Extraction of Polar Metabolites from Mussel Gonads

To obtain polar metabolites with a molecular weight lower than 1.5 KDa, a “two-step” protocol (methanol/chloroform/water) was applied [49]. Therefore, 100 mg of frozen tissue from male and female gonads was collected from each of the specimens taken from each condition (*n* = 9 per sex per experimental condition). The samples were then mixed in cold methanol and distilled water and methanol in the following proportion: water = 4 mL/g: 0.85 mL/g. They were then homogenized in TissueLyser LT (Qiagen, Hilden, Germany) for 10 min with stainless steel beads. Once the homogenate was obtained, 4 mL/g of cold chloroform and 2 mL/g of distilled water were added and mixed manually for 1 min, then left to stand on ice for 10 min. Each sample was placed in a centrifuge refrigerated at 4 °C and centrifuged for 5 min at 2000× *g* to obtain complete separation into three phases and collect only the supernatant containing the polar molecules. With a centrifugal vacuum concentrator (Eppendorf 5301, Milan, Italy), the pellet containing the metabolites was obtained from each sample after a few hours. Before proceeding with the qualitative and quantitative analysis by ^1^H NMR spectrometer, each sample was resuspended in 600 µL of 0.1 M sodium phosphate buffer (pH 7.0, 10% D_2_O; Armar AG, Döttingen, Switzerland) with 1 mM 2,2-dimethyl-2-silapentane-5-sulfonate (DSS; Sigma-Aldrich, USA), which served as a “chemical form indicator” (δ = 0.0 ppm).

#### 2.5.2. Metabolomics Based on ^1^H NMR and Spectral Pre-Processing

For metabolomic analysis, each resuspended sample was inserted into very thin glass capillaries of 5 mm diameter, through which it was possible to introduce the matrix containing the analytes into an NMR spectrometer (Varian-500 NMR; Varian, Inc., Palo Alto, CA, USA) equipped with RF channels that generate wavelengths between 499.74 MHz and 298 K of frequency, a range in which it is possible to detect metabolites with molecular weight lower than 1.5 KDa and of polar character. Using the Nuclear Overhauser Effect SpectroscopY (NOESY) setting that uses the innovative nuclear overhauser effect, implemented with PRE-SAT at a mixing time of 120 ms and a relaxation delay of 2 s for the acquisition of spectral data, very clear and well-resolved NMR spectra were obtained, with a considerable reduction in the interference of the water peak, which also better revealed the metabolites’ resonances within the region between 4.5 and 5.1 ppm. By using the Chenomx Processor software (Chenomx NMR Suite version 12.0 professional; Chenomx Inc., Edmonton, AB, Canada), the baseline was corrected for each spectrum and the DSS was set at 0.0 ppm. Using the Chenomx database and public computer libraries, the identification of each metabolite was performed, whereas their concentration was determined based on the concentration of the DSS used as a standard. Metabolites mainly involved in the energy metabolism (i.e., glucose, glycogen, lactate, acetoacetate) and detoxification (i.e., glycine, glutamate, histidine), as well as other metabolite biomarkers commonly found in mussels [47,49], were the major molecules of interest.

#### 2.5.3. Bioinformatics Analysis on Metabolomic Data

Using the MetaboAnalyst software, version 6.0 (https://www.metaboanalyst.ca) [62], after Pareto scale normalization, Principal Component Analysis (PCA) was performed. This is a chemometric technique used to re-size a data set while maintaining all the main information relative to the data, which proved useful to simplify and give a more intuitive picture of the data collected from the control and PLA BPs groups for both sexes. The principal components were found to maintain the variance (information) and minimize data loss, displaying multivariate NMR information in a 3D score graph in which similar metabolic profiles were grouped together [48,63]. Furthermore, to distinguish the groups from each other (controls and treated male and female gonads, respectively) and to define a classification model, a Partial Least Squares Discriminant Analysis (PLS-DA) was also performed by the MetaboAnalyst metabolomic software. This technique is a variant of PLS, consisting of a multivariate analysis used to find relationships between independent variables and one dependent variable, which returns a classification of the data [64]. Additionally, Variable Importance in Projection (VIP) scores were also applied to evaluate the relative contribution of each predictor variable.

### 2.6. Statistical Analysis

The data obtained from histochemical and biochemical analysis was first processed on Excel (Microsoft 365) to obtain the standard deviation (±SD), and then processed using of the GraphPad Prism software (Prism 8.0). The statistical evaluation was therefore performed using the two-way ANOVA followed by Sidak’s multiple comparisons test for post hoc multiple comparisons, in order to compare mussels from the control versus the PLA BPs groups, considering male and female gonads separately, and considering a *p* value lower than 0.05 (*p* < 0.05) as significant. To further streamline and normalize the data, the average was calculated for each biological replicate. Males and females were then grouped into three sample groups for each replicate. Thus, each dot represented in the graphs corresponds to the average of the samples for each biological replicate.

## 3. Results

### 3.1. Histo-Morphological Observations

**Figure 1:**
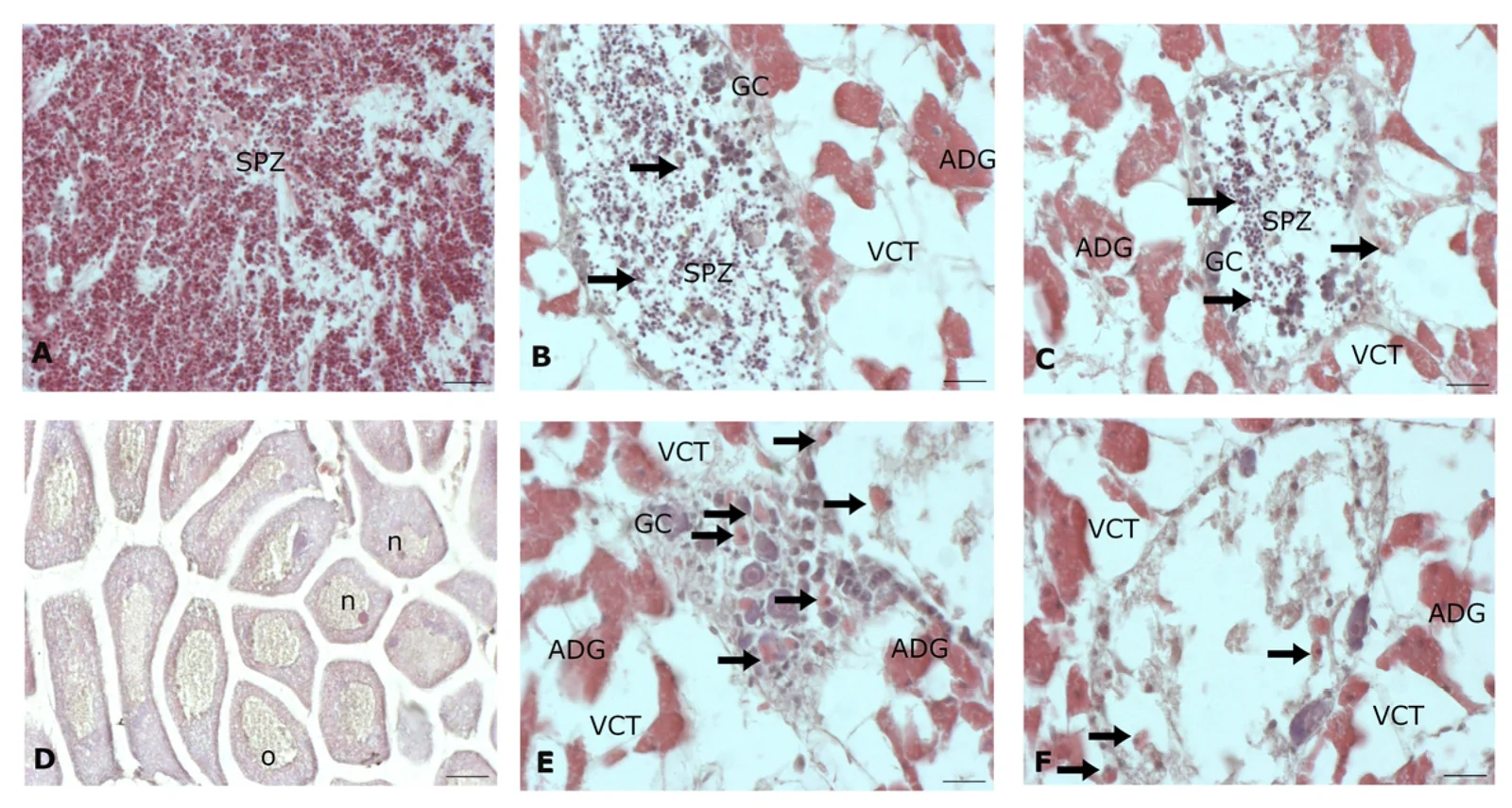
Representative histological images obtained by H/E dichromic staining performed on thin sections of male (**A**–**C**) and female (**D**–**F**) gonadal tissue of *Mytilus galloprovincialis* from the control group (**A**,**D**) and exposed to 0.5 µg/mL PLA BPs (**B**,**C**,**E**,**F**) after seven days of treatment. Spermatozoa (SPZ); germ cells (GC); oocytes (O); nucleolus (n); vesicular connective tissue (VCT); adipogranular cells (ADG); hemocyte infiltration (arrows). 40× magnification; Scale bar: 20 μm.

Using the H/E colorimetric method, it was possible to establish the reproductive period in which the gonad of mussels was collected. In fact, in both sexes there was a typical organization of the gametogenic period, which was consistent with the autumn season in which the experimental plan was carried out (Figure 1). Specifically, the male gonads were found between stage II and IIIA (Figure 1A), while the female ones between were found stage II and III (Figure 1D), with a few exceptions [65,66]. In the male organisms of the control group, the follicles were found towards the lumen filled with spermatozoa, with the heads highlighted in blue-violet by hematoxylin and thin tails highlighted by eosin, while towards the wall few germ cells were distinguishable (Figure 1A). In the group exposed to PLA BPs (Figure 1B,C) for seven days, the male gonadal tissue maintained a structure similar to that shown by mussels from the control group, with follicles with intact walls. However, throughout the connective tissue and inside the follicle, an intense hemocyte recall occurred, resulting in an increased accumulation of hemocyte within the tissue. Signs of early spawning seem to have occurred in some follicles that were found with few disorganized spermatozoa inside (Figure 1B,C).

Even in female gonads of control organisms, follicles were found to be filled with oocytes that had almost reached maturity, in some of which the nucleolus was present, marked in blue-violet compared to the pinker nucleus (Figure 1D). The sections of female gonads treated with PLA BPs still retained a structure like those of the control group; however, the entire tissue, including the connective tissue and the germinal area, was full of hemocytes (Figure 1E,F). As observed in males, in female gonads a general early emptying of the follicles was also observed, some of which retained germinal cells inside them, flanked by the presence of hemocytes that were also inside the follicle (Figure 1E,F).

The original microscopy images are provided in the File S1.

### 3.2. Histochemical Data

**Figure 2:**
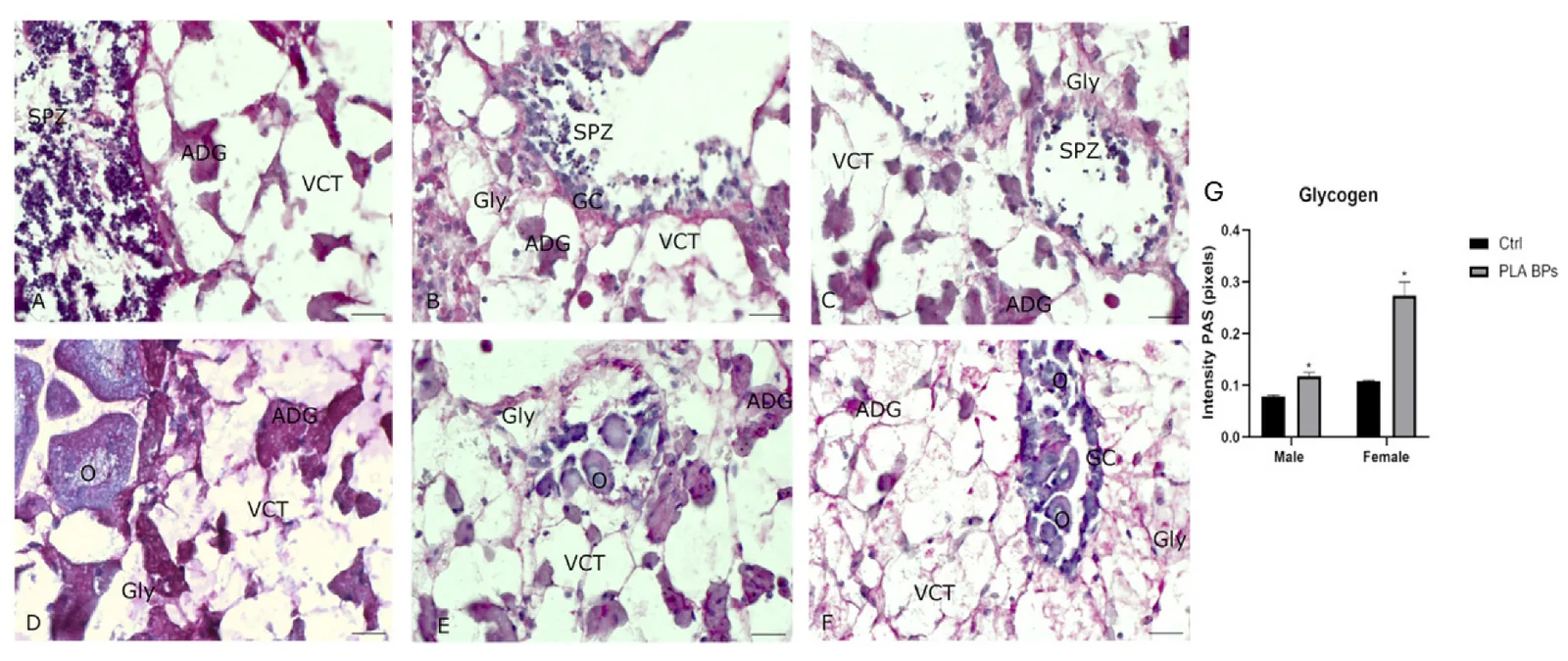
Representative histological images of the d-PAS/PAS reaction on male (**A**–**C**) and female (**D**–**F**) gonadal tissue of *Mytilus galloprovincialis* collected from the control group (**A**,**D**) and exposed to 0.5 µg/mL PLA BPs (**B**,**C**,**E**,**F**) after seven days of treatment. Spermatozoa (SPZ); germ cells (GC); oocytes (O); vesicular connective tissue (VCT); adipogranular cells (ADG); glycogen (Gly). 40× magnification; scale bar: 20 μm. The graph (**G**) shows the intensity of PAS (pixels) representing the average level of glycogen. Asterisks (*) indicate statistically significant differences (*p* < 0.05) between control (Ctrl; black) and PLA BPs group (gray) in male and female mussel gonads (*n* = 9 per sex per experimental condition).

The d-PAS/PAS reactions were performed on separate serial sections of male and female mussel gonads to evaluate the presence of the neutral polysaccharide glycogen and to measure its eventual modulation following the seven-day treatment with 0.5 µg/mL PLA BPs (Figure 2).

The data obtained demonstrated that, different from the male gonads of the control organisms (Figure 2A), the gonads of the treated group showed a slight, but significant increase in positivity to the PAS reaction (Figure 2B,C), exhibiting a magenta color at the level of the vesicular tissue cells (VCT).

Instead, in the female counterpart, it is evident how, compared to gonadal tissues from the control group (Figure 2D), there was a more marked positivity to the PAS reaction in the sections obtained from samples treated with PLA BPs within the VCT (Figure 2E,F).

Furthermore, histochemical analysis demonstrated that there was a clear divergence between the glycogen stores in the connective tissue of male versus female control organisms, and that this disparity increased significantly following seven-day treatment with PLA BPs (Figure 2G).

The original microscopy images are provided in the File S1.

### 3.3. Biochemical Results

**Figure 3:**
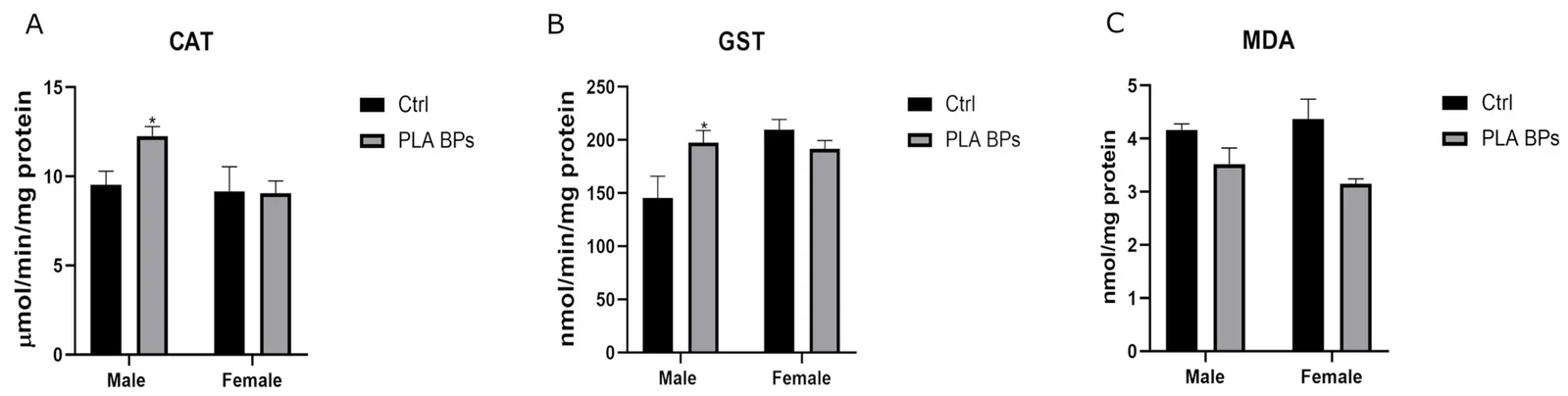
Graphical representation of the enzymatic activity of catalase (CAT, μmol/min/mg of protein) (**A**) and glutathione *S*-transferase (GST; nmol/min/mg protein) (**B**), and concentration of malondialdehyde (MDA, nmol/mg protein) (**C**) in male and female gonads of *Mytilus galloprovincialis* from the control (Ctrl) and exposed to 0.5 µg/mL PLA BPs after seven days of exposure. Asterisks (*) indicate statistically significant differences (*p* < 0.05) between control (Ctrl; black) and PLA BPs group (gray) in male and female mussel gonads (*n* = 9 per sex per experimental condition).

The results obtained from the biochemical analysis carried out on gonadal tissue homogenates of organisms exposed for seven days to PLA BPs did not show any alterations compared to the control group in female mussels with regard to the antioxidant mechanisms represented by the activity of CAT. However, a significant increase (*p* < 0.05) in CAT activity was recorded at the level of the male reproductive system compared to the male control (Figure 3A).

A similar situation was also recorded when evaluating the detoxifying strategies by the enzyme GST, for which a significant increase (*p* < 0.05) in activity was measured only in the male gonads of the treated organisms compared to the control, but not in the female counterpart (Figure 3B).

The data obtained from the biochemical analysis to evaluate the occurrence of LPO at the level of the gonadal tissue of male and female mussels exposed to PLA BPs revealed a slight but not significant reduction in the concentration of MDA compared to the control mussels after the seven-day treatment in both sexes (Figure 3C).

### 3.4. ^1^H NMR Data

Through the qualitative and quantitative analysis of polar metabolites, carried out in mussel gonads by ^1^H NMR spectroscopy, a metabolic profile that was qualitatively similar in composition was found in both sexes, while differences in the concentrations of some metabolites were observed depending on sex. Overall, major alterations were revealed in molecules involved in various metabolic mechanisms, particularly in the energetic metabolism (as suggested by the levels of glucose, glycogen, and lactate) and detoxifying pathways (as indicated by the concentrations of glycine, glutamate, and histidine).

#### 3.4.1. ^1^H NMR Spectra Pattern Recognition Analysis

**Figure 4:**
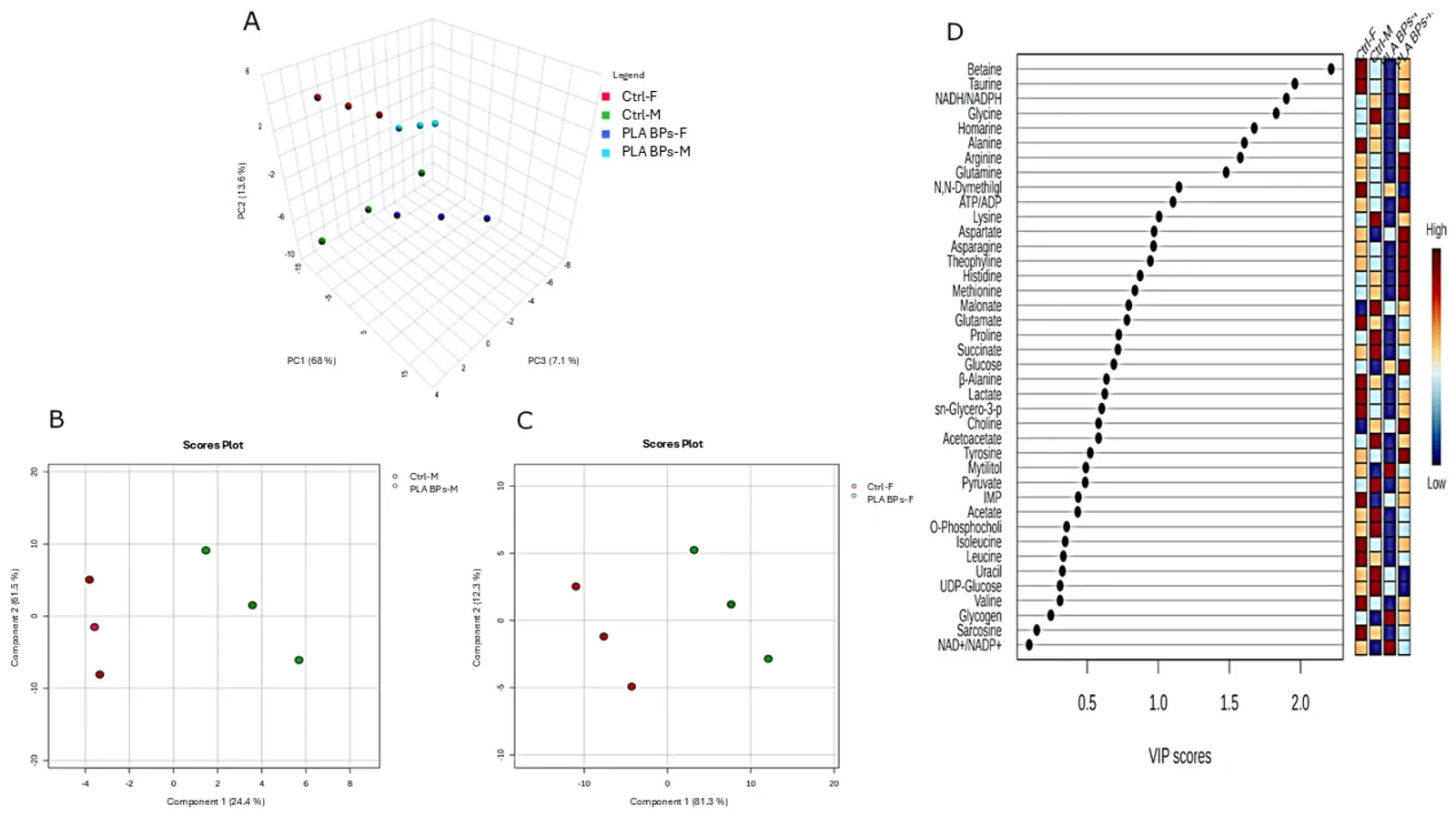
Principal Component Analysis (PCA) (**A**), Partial Least Squares Discriminant Analysis (PLS-DA) (**B**,**C**), and Variable Importance in Projection (VIP) scores (**D**) obtained from metabolomic data based on ^1^H NMR spectrometry of male (M) and female (F) gonads of *Mytilus galloprovincialis* from control (Ctrl) and exposed to 0.5 µg/mL PLA BPs after seven days of exposure (*n* = 9 per sex per experimental condition).

The PCA was performed to determine the metabolic divergences between the control and PLA BPs-treated groups for male and female gonad samples, respectively. The 3D plot of the PCA scores returned the fingerprints of the metabolite profile obtained by ^1^H NMR spectroscopy of the male and female gonads of mussels (Figure 4A), demonstrating a clear difference between the metabolome of control male and female gonads, as well as between control and treatment groups along the PC axes, with 68% variance in PC1, 13.6% in PC2, and 7.1% in PC3.

The PLS-DA, a supervised approach, produced a score plot that could more easily visualize the distribution of the samples in the space of the principal components, clarifying the separation between the different groups of data, specifically between male gonads from the control and treated groups (Figure 4B), and separately between female gonads from the control and treated groups (Figure 4C). In both cases, the two groups were clearly clustered, and therefore the metabolic variables (metabolites) were identified in an exploratory way, allowing for differentiation between the metabolic results for the control and treatment groups of both sexes.

The VIP score obtained by examining samples of both male and female gonads allowed for an evaluation of the importance of each variable (metabolite) in the respective groups, control and treatment with PLA BPs, using a color scale to highlight the weight of the variance in a principal component, and therefore how much a metabolic variable contributes to the separation and classification of the data. A VIP score greater than 1 is commonly used to identify variables that contribute most to class discrimination in the PLS-DA model, while variables with VIP scores less than 1 generally contribute less to class separation. Based on this criterion, some of the metabolites that were most relevant in the distinction between the groups considered (control and treatment) in male and female gonads were identified and examined (Figure 4D). The metabolites responsible for discrimination between the experimental conditions in both sexes are described in detail in the following sub-sections, grouped according to the metabolic pathways in which they are involved.

#### 3.4.2. Energy Metabolism

**Figure 5:**
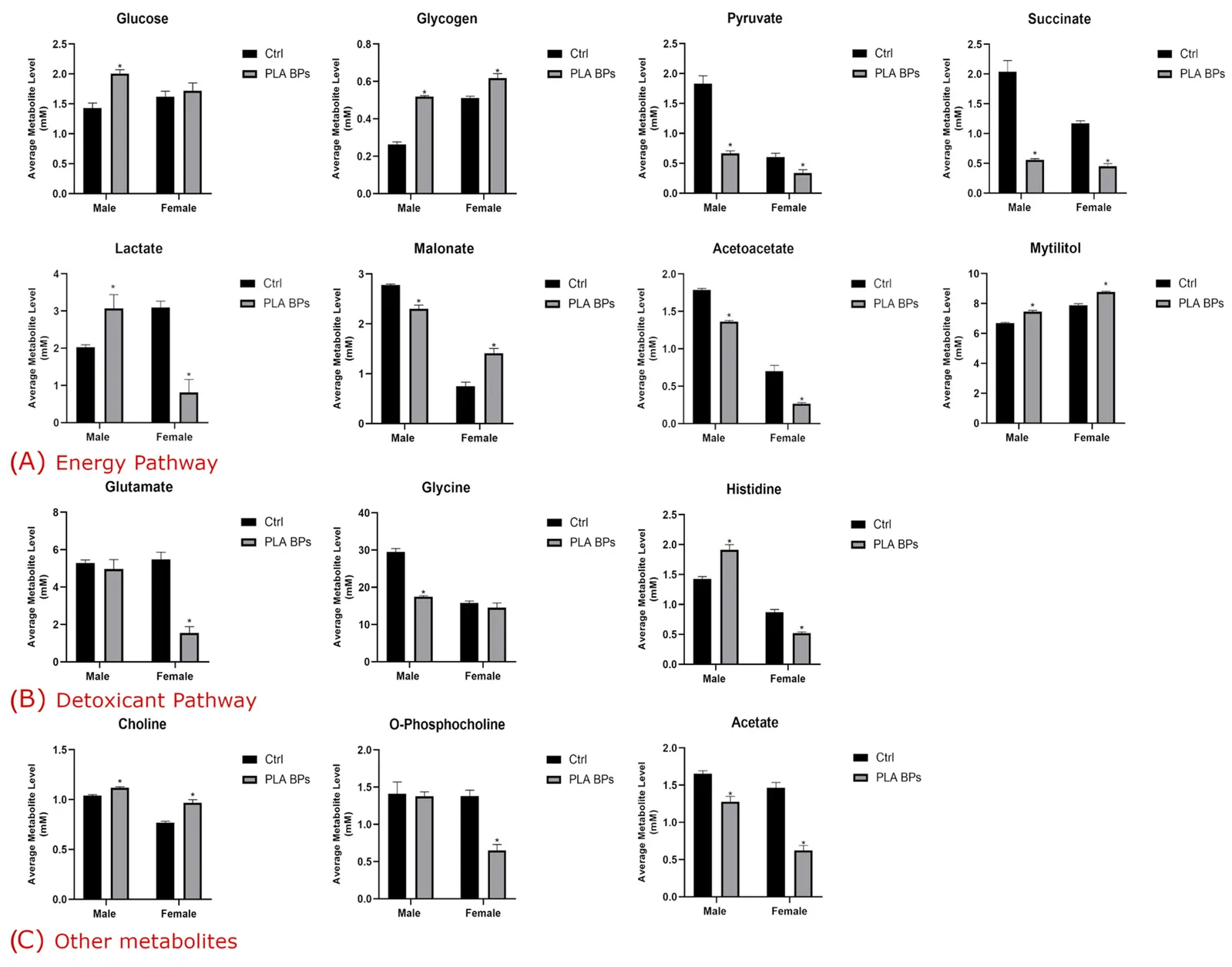
Graphs of the concentrations (mM, mean ± SD) of metabolites involved in the energy metabolism (**A**), detoxificant pathway (**B**), and other metabolites (**C**) measured in male and female gonads of *Mytilus galloprovincialis* from the control group (Ctrl) and those exposed to 0.5 µg/mL PLA BPs after seven days of exposure. Asterisks (*) indicate statistically significant differences (*p* < 0.05) between the control (Ctrl; black) and PLA BPs groups (gray) in male and female mussel gonads (*n* = 9 per sex per experimental condition).

In regard to the metabolites involved in the energy metabolism, in mussel gonads subjected to treatment with PLA BPs, a significant (*p* < 0.05) increase in the level of glucose was recorded solely in male organisms, while in females, its concentration was similar to that of control mussels. Moreover, in regard to the energy reserves represented by glycogen, it was found that they underwent a significant increase in both sexes (Figure 5A). This data indicates an abnormal accumulation of glycogen compared with the control group, and suggests a potential alteration in the physiological energy balance within the gonadal tissue of both sexes.

Metabolites such as pyruvate and succinate showed a significant (*p* < 0.05) decrease in male and female gonads of specimens treated with 0.5 µg/mL of PLA BPs (Figure 5A). Interestingly, a divergent response in the level of lactate was observed, which, in both gonads, showed a significant (*p* < 0.05) alteration, exhibiting an increase in males and a marked decrease in females. This trend was the opposite to that recorded for malonate, which underwent significant depletion in males, unlike the significant elevation observed in female gonads (Figure 5A).

Notably, other metabolites secondarily involved in the energetic mechanism, such as acetoacetate and mytilitol, showed a similar trend between the two sexes, characterized for the former by a significant (*p* < 0.05) decrease compared to the control, and for the latter by a significant increase in the reproductive tissue of both sexes (Figure 5A).

#### 3.4.3. Detoxification Pathway

Regarding metabolites involved in the detoxification pathway, glutamate and glycine underwent a marked and significant (*p* < 0.05) decrease in female and male gonads, respectively, of mussels exposed to PLA BPs for seven days compared to the control group, although their levels were found to overlap with those recorded for glutamate in males and for glycine in females in control mussels (Figure 5B).

Surprisingly, the concentration of the amino acid histidine exhibited a divergent trend in the reproductive tissue of both sexes, showing a significant (*p* < 0.05) increase in males and a significant depletion in females (Figure 5B).

#### 3.4.4. Other Metabolites

Among the additional metabolites found to be altered in mussel gonads following the treatment with PLA BPs for 7 days, a significant (*p* < 0.05) increase compared to the control was found in the level of choline for both sexes. A marked and significant (*p* < 0.05) depletion was instead highlighted for the concentration of O-phosphocholine exclusively in female specimens, whereas it was unchanged in male gonads compared to the to control. Finally, acetate showed a significant (*p* < 0.05) depletion in gonads of both sexes compared to control mussels (Figure 5C).

## 4. Discussion

The disruptive ecological transition led to significant increases in BP production, from 300,000 tons in 2009 to 2.11 million in 2019 [67]. Today, PLA is the most used polymer thanks to its multifunctional properties, with a further growth rate predicted in future [24,68]. This is mainly due to the versatility of the linear aliphatic polyester, which is widely adaptable to various fields of use, from packaging to electro-informatics, and from agriculture to cosmetics [11,14,19,21]. At the international level, the US Food and Drug Administration identified PLA as a promising biopolymer suitable for pharmacological use (such as drug delivery) in the field of medicine and tissue engineering [10,11,24]. The potential capacity of the PLA-based polymer chain to be easily subjected to degradation shows compostability, and would guarantee that it is capable of attenuating and perhaps remedying pollution from conventional plastics, facilitating disposal activities at an industrial level [28].

This idyllic vision, however, is not exactly reality, as the characteristics that make PLA an advantageous polymer also lead to disadvantages. In fact, the mechanisms underlying the degradation and subsequent mineralization under natural conditions require a lot of time and specific chemical–physical conditions and microorganisms capable of producing enzymes, such as proteinase K, are capable of degrading the compound [69]. In addition, it is true that the fragility of the amorphous parts of the biopolymer, combined with the low speed at which the hydrolytic process occurs, requiring temperatures above 55 °C [70], lead to the product persisting in the environment and the production of smaller particles, such as MPs [71]. These observations have smoothed out the gap between the two types of plastic, highlighting that both are easily subjected to the mechanical action of waves, particularly in coastal marine environments, and to the chemical–physical–microbial processes that lead to the inevitable fragmentation of the polymer chain and the ubiquitous dispersion of the BPs that can come into contact with the biota at multiple levels. Furthermore, preliminary data have highlighted how the quality of the bio-composite can make a surface colonizable by biotic components, such as microorganisms, and abiotic ones, such as metals and drugs, raising concerns about the vectorial activity of other substances, which is aggravated by their speed of release once inside the host [72,73].

Bearing in mind the current path undertaken by most countries, mainly European countries, to improve the health of the Earth ecosystem through an ecological transition according to a “One Health” perspective, the aim of this study was to verify the effect of an environmental concentration of PLA biopolymers [33,34,35] on the health of the male and female reproductive system of the Mediterranean mussel *Mytilus galloprovincialis*, following a seven-day exposure.

The data obtained from histological observations by the H/E colorimetric method suggested that short-term exposure is sufficient for PLA BPs to access more internal body districts, such as the gonads. As a consequence of this, compared to control organisms, mussel gonads of both sexes showed an intense hemocyte response. Such an immunological response is also typical of conventional MPs, and seems to be attributable to their steric action. In fact, previous studies have shown that 5 µm PS MPs can trigger a cellular defense in organs that are more frequently in contact with the external aqueous medium, such as the mussel gills, and in more internal organs, such as the digestive gland, even after 48 h [3,48]. This idea is corroborated by a study by [74], which confirms how, after about seven weeks of exposure to PLA microparticles, the bulky biopolymer, causing an abrasion mechanism, was able to induce proteomic modifications at the level of the C1qDC, a component involved in the pathogenic recognition of *Mytilus edulis* hemocytes. These data support that the presence of hemocytes, and can therefore be interpreted as a biomarker of stress. An intense hemocyte call in response to environmental stress is part of the innate immunity strategies typical of bivalves and of the species *M. galloprovincialis* [47,75], suggesting that both conventional and biodegradable plastics can induce a pro-inflammatory condition, as previously reported [3,76].

The idea that a mechanical event stimulates an immune response could also coincide with another effect found in gonads of both organisms after seven days of treatment, i.e., early spawning events. In fact, the encumbrance may have exerted a sort of pressure, stimulating the early release of gametes. Inside the emptied follicles, the remaining gametes can undergo a degenerative process, particularly in female mussels. Otherwise, as happened with conventional plastics, a reallocation of energy resources to the benefit of other districts may occur, due to the reabsorption of energy because of starvation or to an increase in energy demand [77]. This implies, in any case, a more intense hemocyte recall right inside the follicle where the gametes are present. Similarly, alterations in male and female follicles were found in the sea urchin *Paracentrotus lividus* when exposed to various types of bioplastics, including PLA, which induced atretic oocytes at high concentrations [78].

The hypothesis of a reorganization of the energy plan suggests an adaptive strategy is necessary to ensure the maintenance of a certain physiological balance, as documented in the genus *Mytilus* [3]. In this context, the reproductive system can play a crucial role, since the gonad, and particularly the connective tissue, are predisposed to the retention of energy reserves, such as carbohydrates, proteins and lipids [65,66,79]. Since organisms in which fertilization is an external mechanism are more flexible and adaptable [80], it is possible that, like PS MPs, PLA BPs can also influence the bioenergetic system in a similar way, inducing a consequent response [81]. In fact, the data obtained from histochemical analysis showed that, compared to the control organisms of both sexes, treatment with 0.5 µg/mL of PLA BPs promoted an increase in glycogen accumulation in VCT, which is a tissue specifically and structurally predisposed to the accumulation of the racemic polysaccharide, an energy compound that is necessary during the gametogenic period for the maturation of male and female gametes [82,83]. In support of this hypothesis, the results obtained from the metabolomic analysis showed a significant increase in cellular glycogen in both sexes. A study, the measurement of biochemical indicators including glycogen highlighted a similar energetic compensation promoted by aged conventional PS MPs, suggesting that the Pacific oyster redirects its gonadal reserves to the storage of energetic resources in the form of glycogen as a response to a physiological stress induced by the contaminant [84]. Furthermore, in *M. galloprovincialis*, a similar bioenergetic imbalance was also highlighted in the digestive gland that, after a brief exposure of 72 h [48], demonstrated the deposition of the branched polysaccharide, in addition to an accumulation of glucose, an attitude that coincides with what was found in the present study, at least for males. It is likely that since PLA is very similar to conventional polymers such as PS or PET [85], there are some homologies in the effects observed, as supported by [22].

From the trend shown in metabolites such as pyruvate and succinate, it would seem that the normal aerobic pathways for energy production remain solid and intact in both sexes, at least for seven days of exposure. However, the accumulation of lactate in the male gonad, combined with the marked depletion of pyruvate and malonate, leads to the hypothesis that there is an initial influence of the contaminant on the normal energy production cycle that could induce, particularly in males compared to females, the activation of secondary pathways to satisfy the greater energy demand [86]. A first hypothesis for these results could be represented by the role of malonate, which is a competitor of succinate [87,88]. Its depletion would propose there is a steric relationship with the enzyme succinate dehydrogenase, of which it is a competitive inhibitor, which leads to the formation of an obstacle to the normal cycle of tricarboxylic acids, as occurred in the digestive gland of *M. galloprovincialis* exposed to PS MPs [48].

Another perspective would be supported by the trend in lactate, which could act as an important energy substrate mainly at the level of the male reproductive system of the species, as it supports the maturation of germ line cells, such as spermatocytes and spermatids, in addition to playing a protective role against degenerative events such as apoptosis [76,89]. This degenerative condition was confirmed in zebrafish larvae at seven days post-fertilization when exposed to virgin and photo-oxidized PLA BPs [42]. This hypothesis is supported by the increase in C-methyl-scyllo-inositol, better known as mytilitol [90,91], a 7-C sugar alcohol proposed in *M. galloprovincialis* as a source of energy, the increase in which, like that detected in male and female gonads following PLA BPs exposure, is related to a state of energy deficit [47,91,92].

A comparison of the effects detected in similar species is difficult, as knowledge of the presence of these micropollutants is still emerging and not well-defined compared to their known counterparts. However, there are common points between MPs and BPs. According to [43], the toxic impact of PLA is mainly attributable to the interference in redox homeostasis, an action that is not far from that of conventional MPs related to ROS production [93]. This can compromise the antioxidant capacity, causing the activation of strategies to counteract and compensate for possible damage attributable to the energy metabolism, which is the first to be involved in the response to MP exposure [43,94]. This occurs in gonads during the gametogenic phase more than in other areas of the body, and is of fundamental importance [82,83,89]. This hypothesis is corroborated by the metabolic trend of the amino acid histidine, which, together with cysteine, leads to the formation, first catalyzed by the enzyme iron-dependent sulfoxide synthase II (OvoA), of ovothiol or π-N-methyl-5-thiohistidine, a non-enzymatic molecule that acts as a hydrogen peroxide scavenger [95]. This plays a crucial role in bivalves at the level of the reproductive system, during gametogenesis and in the fertilization process [96,97,98]. Ovothiol is credited with a protective role, which is mainly detected in oocytes [95]. This would explain why an intense depletion of histidine, likely due to the formation of ovothiol, was found solely in female gonads compared to control organisms. This also suggests that males and females can implement divergent strategies in response to the same stress factor.

The disturbance of tissue homeostasis affecting the redox balance is supported by the activities of enzymes of the detoxification mechanism, such as GST, and of the antioxidant process, such as CAT. GST is involved in phase II for the conjugation of xenobiotic substances/primary metabolites of phase I with reduced glutathione to make the exogenous molecules more hydrophilic, and therefore more easily disposable, whereas CAT carries out the dismutation (reduction) of hydrogen peroxide into water and molecular oxygen. Both enzymes are excellent biomarkers for the cytotoxic evaluation of the effect of MPs [3,99,100]. The effects of different types of MPs, including PLA, were evaluated on *Dreissena bugensis* for nine days [101], revealing specific responses dependent on the polymer type, with increased CAT activity after treatment with PLA BPs. Organisms at the larval stage of *Tenebrio molitor*, an insect capable of using various types of traditional plastics to feed on, which are digested and then degraded in the intestine, showed the predilection of PLA. However, after six weeks, a diet based on this biopolymer caused an increase in ROS production compared to the control group fed only bran, suggesting that PLA BPs, almost like traditional PS MPs, can cause a disorder in the redox balance [102]. Another study on MPs and BPs, also in combination with cadmium, conducted on *Eisenia fetida* revealed that exposure to PLA alone can stimulate ROS production after a short-term period at higher levels compared to other experimental conditions at seven days [103]. Furthermore, this treatment induced, at least for up to fourteen days, an increase in CAT activity that, as exposure continues, tends to decrease [103]. This seems to be in line with the data obtained from this study, which showed the influence of PLA BPs on the antioxidant system in male gonads, in which CAT and GST activities were positively affected compared to the control. Surprisingly, females do not seem to activate any defense strategy, suggesting that sex can influence the response to the same stress by activating sex-specific metabolic pathways [91,104].

As already stated, it is difficult to compare data from the literature since few studies to date have explored the effects of PLA BPs on the same model organism. However, other authors have demonstrated how organisms of the species *Mytilus edulis* responded in a similar way after eight days of exposure to PLA BPs, reporting slight concentration-dependent alterations (µg/L) in the enzymatic activity of CAT and GST, a sign of an adaptation strategy to compensate for the challenge posed by the micropollutant [105].

These results are also consistent with the results observed by ^1^H NMR spectrometry related to the trends in glycine and glutamate, which are two of the three amino acids, together with cysteine, that constitute the tripeptide glutathione (GSH) [106]. This thiol is a molecule that plays a key role in detoxification processes, but also in the regulation of some cellular mechanisms; in fact, the cellular redox balance between the reduced (GSH) and oxidized (GSSG) forms may depend on its concentration, with latter representing an activation signal for the early pathway of the apoptosis process [107]. Glutathione with the GST enzyme act in phase II of the biotransformation process; in fact, GST conjugates harmful molecules/compounds to GSH to reduce its toxicity [47,108]. For this reason, a variation in the amino acids constituting the non-protein thiol can be understood as a signal of the influence of PLA BPs on the normal redox balance of the gonad in both sexes, in which glycine and glutamate undergo a depletion compared to the control gonads. A similar response was observed for conventional PS MPs at the level of the reproductive tissue, suggesting that a more sustained GSH turnover is essential to counteract the redox imbalance in both sexes [76,86,109,110]. This could lead to damage to the normal maturation process of germ line cells in both sexes at a crucial time, such as gametogenesis, and induce permanent damage to mature gametes that may be transmitted to offspring, as highlighted for PS MPs in other organisms [111,112].

To obtain an overall picture of the oxidative state to which the target organ was subjected, the concentration of MDA in gonads of both sexes was also evaluated, highlighting that there was no variation between the control group and the one treated with 0.5 µg/mL of PLA BPs. The absence of a significant alteration in MDA may reflect the dynamic and compensatory nature of redox regulation and should be interpreted as a disturbance in redox homeostasis rather than as evidence of increased lipid peroxidation per se. This suggested the absence of an oxidative state of the lipid component, a result that differs from what was detected by the metabolomic analysis of the lipidome in *M. edulis*, for which an alteration in the glycerophospholipid component was demonstrated [105]. This result is supported by the decrease in acetoacetate, a ketone body obtained from lipid catabolism [81]. However, its cellular subtraction, in the present context, could be better interpreted as the use of the metabolite as a carbon substrate involved in the aerobic production of energy through the mitochondrial action of the enzyme succinyl-CoA transferase (SCOT) [113].

The analysis of polar metabolites revealed a depletion of choline followed by an increase in O-phosphocholine. The first is involved in various cellular contexts, plays a cooperative role to ensure the stability of the cell membrane, is involved in the metabolism of nutrients and plays a role as a neurotransmitter in the form of acetylcholine [89]. The second is a precursor of phosphatidylcholine, which, together with other phospholipids such as sphingomyelins, is important to the structural maintenance of plasma membranes [114]. The increase in choline could be associated with the action of PLA on the enzyme acetylcholinesterase (AChE), as supported in *M. coruscus*, in which more intense enzymatic activity was highlighted at the level of the gill tissue, associated with a stimulating action correlated with an antioxidant and immunological response [22]. However, this perspective does not seem to perfectly match the finding of an opposite trend in acetate, a metabolite that conjugated to choline and constitutes the neurotransmitter acetylcholine. On the other hand, a decrease in O-phosphocholine was detected. The latter, together with choline kinase, is involved in the metabolism of choline which, together with ATP, is catalyzed, leading to the formation of phosphocholine and ADP [115]. The increased choline and depleted O-phosphocholine suggest that PLA BPs impaired the conversion of choline to O-phosphocholine at the gonad in both sexes. According to [116], this would be further evidence of a bioenergetic perturbation in the organisms of the species in question. Otherwise, the reduction in membrane phospholipid precursors may be related to an induction of ROS formation, which can cause a decrease in phospholipid availability, as mediated by the estrogen metabolism in response to stress conditions [117], with potential implications for reproductive health [118].

A limitation of this study concerns the physico-chemical characterization of PLA particles. The particle size distribution was determined, while other relevant properties, including morphology, zeta potential, and specific surface area, were not experimentally evaluated. These characteristics, together with the dispersion state and potential aggregation of particles in seawater, can influence particle behavior, actual exposure conditions, and interactions with organisms. Specifically, the dispersion state and potential aggregation of particles in seawater could influence their bioavailability, as demonstrated by the literature documenting the complex fate of BPs in marine environments [51]. Furthermore, differences in particle size may also help to contextualize the variability of the effects observed in different studies. Recent evidence from several aquatic organisms, including marine rotifers, shrimps, and fish, has shown that PLA nanoplastics can be dispersed, but have a slight tendency to aggregate, and can accumulate in exposed organisms without inducing significant acute toxic effects [119]. These findings suggest that biological responses to PLA may vary depending on exposure conditions, including particle size, which may therefore contribute to some of the discrepancies reported in the literature. Although size-dependent effects were not specifically investigated in the present study, this aspect should be considered when comparing results between studies and warrants further investigation. In addition, the exposure period adopted (7 days) could also affect particle dispersion, as well as the biological impact on exposed organisms, since adverse effects were documented in marine zooplankton even within 24 h of PLA exposure [120]. Therefore, further studies combining biological endpoints with a detailed physico-chemical characterization of PLA particles under seawater conditions could help to better define the relationship between particle properties and provide a more complete understanding of the mechanisms underlying the observed effects.

## 5. Conclusions

The findings from this work, which presented multiple levels of investigation (histological, histochemical, biochemical and metabolomics), highlighted how in the native Mediterranean species *Mytilus galloprovincialis*, treatment with PLA BPs at environmental concentrations (0.5 µg/mL) for seven days can promote a strong immune response in the male and female reproductive system, accompanied by alterations in the activity of key enzymes in the antioxidant (CAT) and detoxifying (GST) mechanisms. However, it was also revealed that these conditions are not sufficient to induce conspicuous damage to the lipid component in a short period (absence of peroxidation). Overall, it was evident that PLA BPs can modulate the bioenergetic balance, significantly influencing both sexes.

In summary, although the treatment conditions did not cause severe subcellular alterations, early deposition events resulted in the release of immature gametes that were unable to fertilize, thus potentially compromising the success of fertilization and reproductive fitness of the species, suggesting an endocrine-disrupting effect of PLA BPs. However, the presence of germ cells along the follicle walls suggests that proliferative capacity may be preserved in an attempt at tissue reorganization. These findings also indicate that biological responses may vary according to sex and reproductive maturity.

Overall, the limited data currently available are insufficient to draw definitive conclusions on the effects of PLA BPs. Nevertheless, the similarities to conventional plastics that were observed highlight the need for caution, particularly as PLA production and waste are expected to increase. Appropriate waste-management strategies should therefore be implemented to prevent the environmental and health issues associated with conventional plastics, and to support a truly sustainable green economy.

## Data Availability

The original contributions presented in this study are included in the article. Further inquiries can be directed to the corresponding author.

## Supplementary Materials

The following supporting information can be downloaded at: https://www.mdpi.com/article/10.3390/jox16050176/s1, File S1: Original microscopy images.

## Abbreviations

The following abbreviations are used in this manuscript:

BPs	Bioplastics
PLA	Polylactic Acid
^1^H NMR	Proton Nuclear Magnetic Resonance
WWTPs	Wastewater Treatment Plants
FSW	Filtered Sea Water
PFA	Paraformaldehyde
BPS	Phosphate-Buffered Saline
H/E	Hematoxylin/Eosin
d-PAS/PAS	Diastasis-Periodic Acid Shiff/PAS
CAT	Catalase
GST	Glutathione *S*-Transferase
MDA	Malondialdehyde
LPO	Lipid Peroxidation
H_2_O_2_	Hydrogen Peroxide
CDNB	1-Chloro-2,4-Dinitrobenzene
TBARS	Thio-Barbituric Acid Reactive Method
D_2_O	Deuterated Water
DSS	2,2-Dimethyl-2-Silapentane-5-Sulfonate
NOESY	Nuclear Overhauser Effect Spectroscopy
PCA	Principal Component Analysis
PLS-DA	Partial Least Squares Discriminant Analysis
SPZ	Spermatozoa
GC	Germ Cells
O	Oocytes
n	Nucleolus
VCT	Vesicular Connective Tissue
ADG	Adipogranular
Gly	Glycogen
ROS	Reactive Oxygen Species
VIP	Variable Importance in Projection
PS	Polystyrene
PET	Polyethylene Terephthalate
HD-PE	High-density polyethylene
GSH	Glutathione reduced
GSSG	Glutathione oxidized
SCOTT	Succinyl-CoA transferase enzyme
AChE	Acetylcholinesterase
ATP	Adenosine Triphosphate
ADP	Adenosine Diphosphate

## References

[1] Rai P.K., Sonne C., Song H., Kim K.H. (2023). Plastic wastes in the time of COVID-19: Their environmental hazards and implications for sustainable energy resilience and circular bio-economies. Sci. Total Environ..

[2] La Pietra A., Fasciolo G., Lucariello D., Motta C.M., Venditti P., Ferrandino I. (2024). Polystyrene microplastics effects on zebrafish embryological development: Comparison of two different sizes. Environ. Toxicol. Pharmacol..

[3] De Marco G., Eliso M.C., Conti G.O., Galati M., Billè B., Maisano M., Ferrante M., Cappello T. (2023). Short-term exposure to polystyrene microplastics hampers the cellular function of gills in the Mediterranean mussel *Mytilus galloprovincialis*. Aquat. Toxicol..

[4] Del Piano F., Lama A., Piccolo G., Addeo N.F., Iaccarino D., Fusco G., Riccio L., De Biase D., Raso G.M., Meli R. (2023). Impact of polystyrene microplastic exposure on gilthead seabream (*Sparus aurata* Linnaeus, 1758): Differential inflammatory and immune response between anterior and posterior intestine. Sci. Total Environ..

[5] Jalaudin Basha N.N., Adzuan Hafiz N.B., Osman M.S., Abu Bakar N.F. (2023). Unveiling the noxious effect of polystyrene microplastics in aquatic ecosystems and their toxicological behavior on fishes and microalgae. Front. Toxicol..

[6] Lee J.H., Kang J.C., Kim J.H. (2023). Toxic effects of microplastic (polyethylene) on fish: Accumulation, hematological parameters and antioxidant responses in Korean Bullhead, *Pseudobagrus fulvidraco*. Sci. Total Environ..

[7] Eliso M.C., Billè B., De Marco G., Pulvirenti E., Dal Bello F., Rapisarda P., Pereira P., Galati M., Oliveri Conti G., Ferrante M. (2025). Embryotoxicity of polystyrene microplastics, alone and conjugated with bisphenol A, in the black sea urchin *Arbacia lixula*: A multi-biomarker approach. J. Hazard. Mater..

[8] Barone L., Rossi F., Borgese M., Maisano M., Cappello T., Raspanti M., Pagiatakis C., Papait R., Bernardini G., Gornati R. (2026). Health risks of pristine and leached polystyrene micro- and nanoplastics: An in vitro study on human dental pulp stem cells. Microplastics.

[9] Mangal M., Rao C.V., Banerjee T. (2023). Bioplastic: An eco-friendly alternative to non-biodegradable plastic. Polym. Int..

[10] Ahmad A., Banat F., Alsafar H., Hasan S.W. (2024). An overview of biodegradable poly (lactic acid) production from fermentative lactic acid for biomedical and bioplastic applications. Biomass Convers. Biorefin..

[11] Kong U., Mohammad Rawi N.F., Tay G.S. (2023). The potential applications of reinforced bioplastics in various industries: A review. Polymers.

[12] Venkatachalam H., Palaniswamy R. (2020). Bioplastic world: A review. J. Adv. Sci. Res..

[13] Swetha T.A., Ananthi V., Bora A., Sengottuvelan N., Ponnuchamy K., Muthusamy G., Arun A. (2023). A review on biodegradable polylactic acid (PLA) production from fermentative food waste. Its applications and degradation. Int. J. Biol. Macromolec..

[14] Swetha T.A., Bora A., Mohanrasu K., Balaji P., Raja R., Ponnuchamy K., Muthusamy G., Arun A. (2023). A comprehensive review on polylactic acid (PLA). Synthesis, processing and application in food packaging. Int. J. Biol. Macromolec..

[15] Shen M., Hu T., Huang W., Song B., Qin M., Yi H., Zeng G., Zhang Y. (2021). Can incineration completely eliminate plastic wastes? An investigation of microplastics and heavy metals in the bottom ash and fly ash from an incineration plant. Sci. Total Environ..

[16] Ibrahim N.I., Shahar F.S., Sultan M.T.H., Shah A.U.M., Safri S.N.A., Mat Yazik M.H. (2021). Overview of bioplastic introduction and its applications in product packaging. Coatings.

[17] Shah M., Rajhans S., Pandya H.A., Mankad A.U. (2021). Bioplastic for future: A review then and now. World J. Adv. Res. Rev..

[18] European Bioplastics (2023). Bioplastics Market Development Update 2023.

[19] Mehmood A., Raina N., Phakeenuya V., Wonganu B., Cheenkachorn K. (2023). The current status and market trend of polylactic acid as biopolymer: Awareness and needs for sustainable development. Mater. Today Proc..

[20] European Bioplastics (2022). Market—European Bioplastics. https://docs.european-bioplastics.org.

[21] Folino A., Karageorgiou A., Calabrò P.S., Komilis D. (2020). Biodegradation of wasted bioplastics in natural and industrial environments: A review. Sustainability.

[22] Zhong Z., Shang W., Yang P., Wang S., Chen L., Chen Z., Li L., Khalil M.F., Hu M., Xu X. (2024). Bio-based microplastic polylactic acid exerts the similar toxic effects to traditional petroleum-based microplastic polystyrene in mussels. Sci. Total Environ..

[23] Li G., Zhao M., Xu F., Yang B., Li X., Meng X., Teng L., Sun F., Li Y. (2020). Synthesis and biological application of polylactic acid. Molecules.

[24] Balla E., Daniilidis V., Karlioti G., Kalamas T., Stefanidou M., Bikiaris N.D., Vlachopoulos A., Koumentakou I., Bikiaris D.N. (2021). Poly (lactic Acid): A versatile biobased polymer for the future with multifunctional properties—From monomer synthesis, polymerization techniques and molecular weight increase to PLA applications. Polymers.

[25] Cao Y., Zhang B., Song X., Dong G., Zhang Y., Chen B. (2024). Polyhydroxybutyrate Plastics Show Rapid Disintegration and More Straightforward Biogeochemical Impacts than Polyethylene under Marine Biofragmentation. Environ. Sci. Technol..

[26] Urbanek A.K., Strzelecki M.C., Mirończuk A.M. (2021). The potential of cold-adapted microorganisms for biodegradation of bioplastics. Waste Manag..

[27] Barbe V., Jacquin J., Bouzon M., Wolinski A., Derippe G., Cheng J., Cruaud C., Roche D., Fouteau S., Petit J.-L. (2024). Bioplastic degradation and assimilation processes by a novel bacterium isolated from the marine plastisphere. J. Hazard. Mater..

[28] Zaaba N.F., Jaafar M. (2020). A review on degradation mechanisms of polylactic acid: Hydrolytic, photodegradative, microbial, and enzymatic degradation. Polym. Eng. Sci..

[29] Zoungranan Y., Lynda E., Dobi-Brice K.K., Tchirioua E., Bakary C., Yannick D.D. (2020). Influence of natural factors on the biodegradation of simple and composite bioplastics based on cassava starch and corn starch. J. Environ. Chem. Eng..

[30] Ribba L., Lopretti M., de Oca-Vásquez G.M., Batista D., Goyanes S., Vega-Baudrit J.R. (2022). Biodegradable plastics in aquatic ecosystems: Latest findings, research gaps, and recommendations. Environ. Res. Lett..

[31] Chamas A., Moon H., Zheng J., Qiu Y., Tabassum T., Jang J.H., Abu-Omar M., Scott S.L., Suh S. (2020). Degradation rates of plastics in the environment. ACS Sustain. Chem. Eng..

[32] Akdemir T., Gedik K. (2023). Microplastic emission trends in Turkish primary and secondary municipal wastewater treatment plant effluents discharged into the Sea of Marmara and Black Sea. Environ. Res..

[33] Okoffo E.D., Chan C.M., Rauert C., Kaserzon S., Thomas K.V. (2022). Identification and quantification of micro-bioplastics in environmental samples by pyrolysis–gas chromatography–mass spectrometry. Environ. Sci. Technol..

[34] An G., Na J., Song J., Jung J. (2024). Chronic toxicity of biodegradable microplastic (Polylactic acid) to *Daphnia magna*: A comparison with polyethylene terephthalate. Aquat. Toxicol..

[35] Yan X., Chen Q., Zhang Z., Fu Y., Huo Z., Wu Y., Shi H. (2023). Chemical features and biological effects of degradation products of biodegradable plastics in simulated small waterbody environment. Sci. Total Environ..

[36] Zimmermann L., Göttlich S., Oehlmann J., Wagner M., Völker C. (2020). What are the drivers of microplastic toxicity? Comparing the toxicity of plastic chemicals and particles to *Daphnia magna*. Environ. Pollut..

[37] Emadian S.M., Onay T.T., Demirel B. (2017). Biodegradation of bioplastics in natural environments. Waste Manag..

[38] Karamanlioglu M., Preziosi R., Robson G.D. (2017). Abiotic and biotic environmental degradation of the bioplastic polymer poly (lactic acid): A review. Polym. Degrad. Stabil..

[39] Duan Z., Chen Y., Dou Y., Fan H., Wang J., Cong J., Sun H., Wang L. (2024). Plastic food? Energy compensation of zebrafish (*Danio rerio*) after long-term exposure to polylactic acid biomicroplastics. J. Hazard. Mater..

[40] Rashid E., Hussain S.M., Ali S., Sarker P.K., Farah M.A. (2024). Investigating the toxicity of polylactic acid microplastics on the health and physiology of freshwater fish, *Cirrhinus mrigala*. Ecotoxicology.

[41] Chagas T.Q., Freitas I.N., Montalvão M.F., Nobrega R.H., Machado M.R.F., Charlie-Silva I., Araújo A.P.d.C., Guimarães A.T.B., Alvarez T.G.d.S., Malafaia G. (2021). Multiple endpoints of polylactic acid biomicroplastic toxicity in adult zebrafish (*Danio rerio*). Chemosphere.

[42] Zhang X., Xia M., Su X., Yuan P., Li X., Zhou C., Wan Z., Zou W. (2021). Photolytic degradation elevated the toxicity of polylactic acid microplastics to developing zebrafish by triggering mitochondrial dysfunction and apoptosis. J. Hazard. Mater..

[43] Zhang L., Luo Y., Zhang Z., Pan Y., Li X., Zhuang Z., Li J., Luo Q., Chen X. (2024). Enhanced reproductive toxicity of photodegraded polylactic acid microplastics in zebrafish. Sci. Total Environ..

[44] Shao Y., Wang Y., Hua X., Li Y., Wang D. (2023). Polylactic acid microparticles in the range of μg/L reduce reproductive capacity by affecting the gonad development and the germline apoptosis in *Caenorhabditis elegans*. Chemosphere.

[45] Anderson G., Shenkar N. (2021). Potential effects of biodegradable single-use items in the sea: Polylactic acid (PLA) and solitary ascidians. Environ. Pollut..

[46] Miglioli A., Tredez M., Boosten M., Sant C., Carvalho J.E., Dru P., Canesi L., Schubert M., Dumollard R. (2024). The Mediterranean mussel *Mytilus galloprovincialis*: A novel model for developmental studies in mollusks. Development.

[47] De Marco G., Cristaldi A., Eliso M.C., Conti G.O., Galati M., Billè B., Terranova M., Parrino V., Cappello T., Ferrante M. (2025). Cellular pathway disturbances elicited by realistic dexamethasone concentrations in gills of mussel *Mytilus galloprovincialis* as assessed by a multi-biomarker approach. Environ. Toxicol. Pharmacol..

[48] Cappello T., De Marco G., Oliveri Conti G., Giannetto A., Ferrante M., Mauceri A., Maisano M. (2021). Time-dependent metabolic disorders induced by short-term exposure to polystyrene microplastics in the Mediterranean mussel *Mytilus galloprovincialis*. Ecotoxicol. Environ. Saf..

[49] Cappello T., Giannetto A., Parrino V., Maisano M., Oliva S., De Marco G., Guerriero G., Mauceri A., Fasulo S. (2018). Baseline levels of metabolites in different tissues of mussel *Mytilus galloprovincialis* (Bivalvia: *Mytilidae*). Comp. Biochem. Physiol. Part D Genom. Proteom..

[50] Schneider C.A., Rasband W.S., Eliceiri K.W. (2012). NIH Image to ImageJ: 25 years of image analysis. Nat. Methods.

[51] Dara M., Torregrossa N., La Corte C., Bisanti L., Bertini F., Parrinello D., Parisi M.G., Cammarata M. (2024). Bioplastics and marine invertebrates: Assessing immunological and environmental impacts in the transition from petrochemical to sustainable polymers. Invert. Surv. J..

[52] Meyerholz D.K., Beck A.P., Goeken J.A., Leidinger M.R., Ofori-Amanfo G.K., Brown H.C., Businga T.R., Stoltz D.A., Reznikov L.R., Flaherty H.A. (2018). Glycogen depletion can increase the specificity of mucin detection in airway tissues. BMC Res. Notes.

[53] Maisano M., Cappello T., Natalotto A., Vitale V., Parrino V., Giannetto A., Oliva S., Mancini G., Cappello S., Mauceri A. (2017). Effects of petrochemical contamination on caged marine mussels using a multi-biomarker approach: Histological changes, neurotoxicity and hypoxic stress. Mar. Environ. Res..

[54] Mai N.T.Q., Batjargal U., Kim W.S., Kim J.H., Park J.W., Kwak I.S., Moon B.S. (2023). Microplastic induces mitochondrial pathway mediated cellular apoptosis in mussel (*Mytilus galloprovincialis*) via inhibition of the AKT and ERK signaling pathway. Cell Death Discov..

[55] Chen Z., Sandoval K., Dean M. (2022). Endometrial glycogen metabolism during early pregnancy in mice. Mol. Reprod. Dev..

[56] Jones C.P., Gelais A.T.S., Byron C.J., Costa-Pierce B.A., Smolowitz R.M., Condon M.E., Parker K.E., Jane A.E., Shippey E.G. (2021). A Histopathological–Biochemical Health Assessment of Blue Mussel *Mytilus edulis*. J. Shellfish Res..

[57] Maisano M., Natalotto A., Cappello T., Giannetto A., Oliva S., Parrino V., Sanfilippo M., Mauceri A. (2016). Influences of environmental variables on neurotransmission, oxidative system, and hypoxia signaling on two clam species from a Mediterranean coastal lagoon. J. Shellfish Res..

[58] Bradford M.M. (1976). A rapid and sensitive method for the quantitation of microgram quantities of protein utilizing the principle of protein-dye binding. Anal. Biochem..

[59] Sureda A., Box A., Tejada S., Blanco A., Caixach J., Deudero S. (2011). Biochemical responses of *Mytilus galloprovincialis* as biomarkers of acute environmental pollution caused by the Don Pedro oil spill (Eivissa Island, Spain). Aquat. Toxicol..

[60] Habig W.H., Pabst M.J., Jakoby W.B. (1974). Glutathione *S*-transferases: The first enzymatic step in mercapturic acid formation. J. Biol. Chem..

[61] Botsoglou N.A., Fletouris D.J., Papageorgiou G.E., Vassilopoulos V.N., Mantis A.J., Trakatellis A.G. (1994). Rapid, sensitive, and specific thiobarbituric acid method for measuring lipid peroxidation in animal tissue, food, and feedstuff samples. J. Agric. Food Chem..

[62] Chong J., Wishart D.S., Xia J. (2019). Using MetaboAnalyst 4.0 for comprehensive and integrative metabolomics data analysis. Curr. Protoc. Bioinform..

[63] Nguyen T.V., Alfaro A.C., Young T., Ravi S., Merien F. (2019). Tissue-specific immune responses to *Vibrio* sp. infection in mussels (*Perna canaliculus*): A metabolomics approach. Aquaculture.

[64] Dumas T., Bonnefille B., Gomez E., Boccard J., Castro N.A., Fenet H., Courant F. (2020). Metabolomics approach reveals disruption of metabolic pathways in the marine bivalve *Mytilus galloprovincialis* exposed to a WWTP effluent extract. Sci. Total Environ..

[65] Prisco M., Agnese M., De Marino A., Andreuccetti P., Rosati L. (2017). Spermatogenic cycle and steroidogenic control of spermatogenesis in *Mytilus galloprovincialis* collected in the Bay of Naples. Anatom. Rec..

[66] Rosati L., Agnese M., Abagnale L., Aniello F., Andreuccetti P., Prisco M. (2019). The Mussel *Mytilus galloprovincialis* in the Bay of Naples: New insights on oogenic cycle and its hormonal control. Anatom. Rec..

[67] Ncube L.K., Ude A.U., Ogunmuyiwa E.N., Zulkifli R., Beas I.N. (2020). Environmental impact of food packaging materials: A review of contemporary development from conventional plastics to polylactic acid based materials. Materials.

[68] Jasrotia S., Gupta S., Puttaiahgowda Y.M., Baborski A. (2026). Recent advances in polylactic acid (PLA)-based sustainable food packaging: A comprehensive review. Appl. Food Res..

[69] Weinstein J.E., Dekle J.L., Leads R.R., Hunter R.A. (2020). Degradation of bio-based and biodegradable plastics in a salt marsh habitat: Another potential source of microplastics in coastal waters. Mar. Pollut. Bull..

[70] Rosli N.A., Karamanlioglu M., Kargarzadeh H., Ahmad I. (2021). Comprehensive exploration of natural degradation of poly (lactic acid) blends in various degradation media: A review. Int. J. Biol. Macromol..

[71] Kliem S., Kreutzbruck M., Bonten C. (2020). Review on the biological degradation of polymers in various environments. Materials.

[72] González-Pleiter M., Pedrouzo-Rodríguez A., Verdú I., Leganés F., Marco E., Rosal R., Fernández-Piñas F. (2021). Microplastics as vectors of the antibiotics azithromycin and clarithromycin: Effects towards freshwater microalgae. Chemosphere.

[73] Liao Y.L., Yang J.Y. (2020). Microplastic serves as a potential vector for Cr in an in-vitro human digestive model. Sci. Total Environ..

[74] Green D.S., Colgan T.J., Thompson R.C., Carolan J.C. (2019). Exposure to microplastics reduces attachment strength and alters the haemolymph proteome of blue mussels (*Mytilus edulis*). Environ. Pollut..

[75] Ayhan M.M., Katalay S., Günal A.Ç. (2021). How pollution effects the immune systems of invertebrate organisms (*Mytilus galloprovincialis* Lamark, 1819). Mar. Pollut. Bull..

[76] Chianese T., Galati M., Cappello T., Maisano M., Lettieri G., Marinaro C., Piscopo M., Fasciolo G., Gravato C., Napolitano G. (2026). Exploring the impact of polystyrene microplastic beads on male gonads of the marine mussel, *Mytilus galloprovincialis*. Environ. Toxicol..

[77] Choi J.S., Kim K., Park K., Park J.W. (2022). Long-term exposure of the Mediterranean mussels, *Mytilus galloprovincialis* to polyethylene terephthalate microfibers: Implication for reproductive and neurotoxic effects. Chemosphere.

[78] Viel T., Cocca M., Esposito R., Amato A., Russo T., Di Cosmo A., Polese G., Manfra L., Libralato G., Zupo V. (2024). Effect of biodegradable polymers upon grazing activity of the sea urchin *Paracentrotus lividus* (Lmk) revealed by morphological, histological and molecular analyses. Sci. Total Environ..

[79] Duinker A., Håland L., Hovgaard P., Mortensen S. (2008). Gonad development and spawning in one and two year old mussels (*Mytilus edulis*) from Western Norway. J. Mar. Biol. Assoc. U. K..

[80] Danton E., Kiyomoto M., Komaru A., Wada K.T., Awaji M., Mathieu M. (1996). Comparative analysis of storage tissue and insulin-like neurosecretion in diploid and triploid mussels *Mytilus galloprovincialis* LMK in relation to their gametogenesis cycle. Invertebr. Reprod. Dev..

[81] Shang Y., Wang X., Chang X., Sokolova I.M., Wei S., Liu W., Fang J.K.H., Hu M., Huang W., Wang Y. (2021). The effect of microplastics on the bioenergetics of the mussel *Mytilus coruscus* assessed by cellular energy allocation approach. Front. Mar. Sci..

[82] Pipe R.K. (1987). Oogenesis in the marine mussel *Mytilus edulis*: An ultrastructural study. Mar. Biol..

[83] Gabbott P.A., Whittle M.A. (1986). Glycogen synthetase in the sea mussel *Mytilus edulis* L.—II. Seasonal changes in glycogen content and glycogen synthetase activity in the mantle tissue. Comp. Biochem. Physiol. Part B Comp. Biochem..

[84] Dong M., Song H., Xie C., Zhang Y., Huang H., Zhang H., Wei L., Wang X. (2024). Polystyrene microplastics photo-aged under simulated sunlight influences gonadal development in the Pacific oyster. Mar. Environ. Res..

[85] Alaraby M., Abass D., Farre M., Hernández A., Marcos R. (2024). Are bioplastics safe? Hazardous effects of polylactic acid (PLA) nanoplastics in *Drosophila*. Sci. Total Environ..

[86] Chianese T., Galati M., Cappello T., Maisano M., Balsamo S., Locascio A., Rosati L., Scudiero R. (2026). Polystyrene microplastic exposure adversely affects oocyte quality and ovary health status in *Mytilus galloprovincialis*. Int. J. Mol. Sci..

[87] Lisi A.D., Prato E., Biandolino F., Sarli G., Negro D., Piana G.L., Marzulli D. (2013). Hepatopancreas mitochondria of *Mytilus galloprovincialis*: Effect of zinc ions on mitochondrial bioenergetics and metabolism. Turk. J. Biol..

[88] Kim Y.S. (2002). Malonate metabolism: Biochemistry, molecular biology, physiology, and industrial application. BMB Rep..

[89] Lettieri G., Marinaro C., Brogna C., Montano L., Lombardi M., Trotta A., Troisi J., Piscopo M. (2023). A Metabolomic Analysis to Assess the Responses of the Male Gonads of *Mytilus galloprovincialis* after Heavy Metal Exposure. Metabolites.

[90] Aru V., Motawie M.S., Khakimov B., Sørensen K.M., Møller B.L., Engelsen S.B. (2020). First-principles identification of C-methyl-scyllo-inositol (mytilitol)–A new species-specific metabolite indicator of geographic origin for marine bivalve molluscs (*Mytilus* and *Ruditapes* spp.). Food Chem..

[91] Galati M., Oueldi M.A., De Marco G., Caruso N., Romeo M., Fazio A., Parrino V., Cappello T., Maisano M. (2026). Global warming and biological adaptation: Thermal priming buffers the impact of heatwaves on the reproductive health of the Mediterranean mussel *Mytilus galloprovincialis*. Mar. Pollut. Bull..

[92] Frizzo R., Bortoletto E., Riello T., Leanza L., Schievano E., Venier P., Mammi S. (2021). NMR metabolite profiles of the bivalve mollusc *Mytilus galloprovincialis* before and after immune stimulation with *Vibrio splendidus*. Front. Molec. Biosci..

[93] Liu T., Hou B., Wang Z., Yang Y. (2022). Polystyrene microplastics induce mitochondrial damage in mouse GC-2 cells. Ecotoxicol. Environ. Saf..

[94] Eliso M.C., Billè B., Cappello T., Maisano M. (2024). Polystyrene micro- and nanoplastics (PS MNPs): A review of recent advances in the use of -omics in PS MNP toxicity studies on aquatic organisms. Fishes.

[95] Diaz de Cerio O., Reina L., Squatrito V., Etxebarria N., Gonzalez-Gaya B., Cancio I. (2020). Gametogenesis-related fluctuations in ovothiol levels in the mantle of mussels from different estuaries: Fighting oxidative stress for spawning in polluted waters. Biomolecules.

[96] Murano C., Zuccarotto A., Leone S., Sollitto M., Gerdol M., Castellano I., Palumbo A. (2022). A survey on the distribution of ovothiol and ovoA gene expression in different tissues and cells: A comparative analysis in sea urchins and mussels. Mar. Drugs.

[97] Castellano I., Seebeck F.P. (2018). On ovothiol biosynthesis and biological roles: From life in the ocean to therapeutic potential. Nat. Prod. Rep..

[98] Wong J.L., Créton R., Wessel G.M. (2004). The oxidative burst at fertilization is dependent upon activation of the dual oxidase Udx1. Dev. Cell.

[99] Auguste M., Leonessi M., Bozzo M., Risso B., Cutroneo L., Prandi S., Kokalj A.J., Drobne D., Canesi L. (2023). Multiple responses of *Mytilus galloprovincialis* to plastic microfibers. Sci. Total Environ..

[100] Abouda S., Galati M., Oliveri Conti G., Cappello T., Abelouah M.R., Romdhani I., Ait Alla A., Ferrante M., Maisano M., Banni M. (2024). Metabolomic and biochemical disorders reveal the toxicity of environmental microplastics and benzo[a]pyrene in the marine polychaete *Hediste diversicolor*. J. Hazard. Mater..

[101] Brehm J., Wilde M.V., Reiche L., Leitner L.C., Petran B., Meinhart M., Wieland S., Ritschar S., Schott M., Boos J.-P. (2022). In-depth characterization revealed polymer type and chemical content specific effects of microplastic on *Dreissena bugensis*. J. Hazar. Mater..

[102] Peng B.Y., Sun Y., Li P., Yu S., Xu Y., Chen J., Zhou X., Wu W.-M., Zhang Y. (2023). Biodegradation of polyvinyl chloride, polystyrene, and polylactic acid microplastics in *Tenebrio molitor* larvae: Physiological responses. J. Environ. Manag..

[103] Shang G., Zhai J., Xu G., Wang L., Wang X. (2023). Ecotoxicological effects of co-exposure biodegradable microplastics polylactic acid with cadmium are higher than conventional microplastics polystyrene with cadmium on the earthworm. Sci. Total Environ..

[104] Blanco-Rayón E., Ivanina A.V., Sokolova I.M., Marigómez I., Izagirre U. (2020). Sex and sex-related differences in gamete development progression impinge on biomarker responsiveness in sentinel mussels. Sci. Total Environ..

[105] Khalid A., Zalouk-Vergnoux A., Benali S., Mincheva R., Raquez J.M., Bertrand S., Poirier L. (2021). Are bio-based and biodegradable microplastics impacting for blue mussel (*Mytilus edulis*)?. Mar. Pollut. Bull..

[106] Billè B., Spagnuolo D., De Marco G., Terranova M., Galati M., Cappello T., Genovese G., Maisano M. (2026). The fungicide thiram alters the embryo-larval development of the black sea urchin *Arbacia lixula* by inducing multifactorial toxicological responses. Environ. Res..

[107] Circu M.L., Yee Aw T. (2008). Glutathione and apoptosis. Free Radic. Res..

[108] Afsa S., De Marco G., Cristaldi A., Giannetto A., Galati M., Billè B., Oliveri Conti G., ben Mansour H., Ferrante M., Cappello T. (2023). Single and combined effects of caffeine and salicylic acid on mussel *Mytilus galloprovincialis*: Changes at histomorphological, molecular and biochemical levels. Environ. Toxicol. Pharmacol..

[109] Mottola F., Carannante M., Barretta A., Palmieri I., Rocco L. (2024). Reproductive cytotoxic and genotoxic impact of polystyrene microplastic on *Paracentrotus lividus* spermatozoa. Curr. Res. Toxicol..

[110] Qiang L., Cheng J. (2021). Exposure to polystyrene microplastics impairs gonads of zebrafish (*Danio rerio*). Chemosphere.

[111] Luo T., Wang C., Pan Z., Jin C., Fu Z., Jin Y. (2019). Maternal polystyrene microplastic exposure during gestation and lactation altered metabolic homeostasis in the dams and their F1 and F2 offspring. Environ. Sci. Technol..

[112] Sun S., Jin Y., Luo P., Shi X. (2022). Polystyrene microplastics induced male reproductive toxicity and transgenerational effects in freshwater prawn. Sci. Total Environ..

[113] Tanaka H., Takahashi T., Iguchi N., Kitamura K., Miyagawa Y., Tsujimura A., Matsumiya K., Okuyama A., Nishimune Y. (2004). Ketone bodies could support the motility but not the acrosome reaction of mouse sperm. Int. J. Androl..

[114] Cappello T., Brandão F., Guilherme S., Santos M.A., Maisano M., Mauceri A., Canario J., Pacheco M., Pereira P. (2016). Insights into the mechanisms underlying mercury-induced oxidative stress in gills of wild fish *Liza aurata* combining ^1^H NMR metabolomics and conventional biochemical assays. Sci. Total Environ..

[115] Pomfret E.A., Schurman L.L., Zeisel S.H. (1989). Measurement of choline and choline metabolite concentrations using high-pressure liquid chromatography and gas chromatography-mass spectrometry. Anal. Biochem..

[116] Wu H., Xu L., Yu D., Ji C. (2017). Differential metabolic responses in three life stages of mussels *Mytilus galloprovincialis* exposed to cadmium. Ecotoxicology.

[117] Rodrigues J.A., Bispo D.S., Silva M.G., Araújo R., Soares A.M., Freitas R., Gil A.M. (2023). Impact of Sea Warming and 17-α-Ethinylestradiol Exposure on the Lipid Metabolism of *Ruditapes philippinarum* Clams. Int. J. Molec. Sci..

[118] Wang Y., Shi Q., Zhang M., Xu L., Wei Q., Zhang R., Sun A., Lu Y., Zhang Z., Shi X. (2024). Combined ecotoxicity of polystyrene micro-plastics and Di-(2-ethylhexyl) phthalate increase exposure risks to *Mytilus coruscus* based on the bioaccumulation, oxidative stress, metabolic profiles, and nutritional interferences. J. Hazard. Mater..

[119] Mustapha D.S., Rodríguez-Díaz O., Cajaraville M.P., Orbea A. (2025). PLA Nanoplastics Accumulate but Do Not Cause Acute Toxicity to Marine Rotifers, Brine Shrimps, and Zebrafish Embryos. J. Xenobiotics.

[120] Di Giannantonio M., Gambardella C., Miroglio R., Costa E., Sbrana F., Smerieri M., Carraro G., Utzeri R., Faimali M., Garaventa F. (2022). Ecotoxicity of Polyvinylidene Difluoride (PVDF) and Polylactic Acid (PLA) Microplastics in Marine Zooplankton. Toxics.

